# Maternal health among Venezuelan women migrants at the border of Brazil

**DOI:** 10.1186/s12889-020-09912-x

**Published:** 2020-11-23

**Authors:** L. Bahamondes, M. Laporte, D. Margatho, H. S. F. de Amorim, C. Brasil, C. M. Charles, A. Becerra, M. M. Hidalgo

**Affiliations:** 1 Department of Obstetrics and Gynaecology, Faculty of Medical Sciences, University of Campinas (UNICAMP), Caixa Postal 6181, Campinas, SP 13084-971 Brazil; 2 Direction of Basic Attention Care, Health Secretary, State of Roraima, Boa Vista, RR Brazil; 3 Direction of Basic Attention Care, Health Secretary, Municipality of Boa Vista, Boa Vista, RR Brazil

**Keywords:** Migrants, Venezuela, Brazil, Sexual and reproductive health

## Abstract

**Background:**

Guaranteeing the sexual and reproductive health and rights (SRHR) of populations living in fragile and humanitarian settings is essential and constitutes a basic human right. Compounded by the inherent vulnerabilities of women in crises, substantial complications are directly associated with increased risks of poor SRHR outcomes for displaced populations. The migration of Venezuelans, displaced due to current economic circumstances, is one of the largest in Latin America’s history. This study aims to provide an overview of the sexual and reproductive health (SRH) issues affecting migrant Venezuelan women in the state of Roraima, Brazil.

**Methods:**

Face-to-face interviews were conducted from 24 to 30 November 2019. Data collection covered various issues involving access to and use of SRH services by 405 migrant Venezuelan women aged 18–49 years. The Minimum Initial Service Package readiness assessment tools, available from the Inter-Agency Working Group on Reproductive Health in Crises, were used in the data collection.

**Results:**

Most commonly, the women reported unmet family planning needs. Of these, a significant proportion reported being unable to obtain contraceptive methods, particularly long-acting reversible contraceptives, either due to the woman’s inability to access them or their unavailability at healthcare centres. Although a significant proportion of women were largely satisfied with the attention received at the maternity hospital, both before and during childbirth, 24.0% of pregnant or postpartum women failed to receive any prenatal or postnatal care.

**Conclusion:**

Meeting the essential SRHR needs of migrant Venezuelan women in Roraima, Brazil is a challenge that has yet to be fully addressed. Given the size of this migrant population, the Brazilian healthcare system has failed to adapt sufficiently to meet their needs; however, problems with healthcare provision are similar for migrants and Brazilian citizens. Efforts need to be encouraged not only in governmental health sectors, but also with academic, non-governmental and international organisations, including a coordinated approach to ensure a comprehensive SRHR response. Given the current high risks associated with the SARS-CoV-2 pandemic, meeting the SRHR needs of migrant populations has become more critical than ever.

## Background

Assuring the sexual and reproductive health and rights (SRHR) of populations living in fragile and humanitarian settings is an essential part of guaranteeing human rights [1]. Over 70.8 million people are estimated to have been forcibly displaced worldwide [2], with an estimated 34 million of these individuals being adolescent girls and women of reproductive age, constituting a significant proportion of displaced populations [2, 3]. Compounded by the inherent vulnerabilities of women in crises, there are other substantial complications that are directly associated with increasing the risks of poor SRHR outcomes for displaced populations. These are often associated with increased rates of exposure to gender-based violence [4], complications during pregnancy and childbirth, unsafe abortions and increased rates of reproductive tract infections, among others [5–11].

Venezuela has been facing a complex economic situation and bearish markets since 2014 [12]. This has translated into deteriorating SRHR indicators among Venezuelan women. Last available estimates showed higher maternal mortality ratios and increased rates of adolescent pregnancy. Moreover, there have been increased rates of HIV infection, with limited availability of antiretrovirals [13, 14] and a lack of effective prevention of mother-to-child transmission of HIV and congenital syphilis [15–17]. The country has also been facing the resurgence of multiple vaccine-preventable diseases (mumps, tetanus, diphtheria, measles and poliomyelitis) and of vector-borne infections (dengue, chikungunya, Zika, malaria) that pose health risks to the population at the borders [18, 19], particularly women, infants and children.

A significant number of Venezuelans are leaving the country, representing the largest displacement in the history of Latin America [20]. Estimates predict that over 289,000 Venezuelans moved to Brazil between 2017 and 2019 [20, 21], particularly through the isolated northern state of Roraima where approximately 40,000 Venezuelans are estimated to be living in the cities of Boa Vista and Pacaraima, representing around 10% of the local population. Thirteen United Nations High Commissioner for Refugees (UNHCR) shelters were set up (including two exclusively for indigenous people) in collaboration with the Brazilian army. The Brazilian Ministry of Health, with help from the Pan American Health Organisation/World Health Organisation (PAHO/WHO), has been conducting mass vaccination campaigns since early 2018 in addition to providing support to strengthen local healthcare and services [22].

To the best of our knowledge, comprehensive data on the SRHR needs of migrant Venezuelan women at the borders are limited. Little is known regarding the availability and delivery of sexual and reproductive health (SRH) services and women’s ability to access these services. Obtaining data is an essential step in improving SRHR response, strengthening SRH services, improving quality and delivery, and meeting current needs. Previous reports indicated that Venezuelan women face multiple barriers that limit their access to SRH services in host countries. These barriers include language difficulties, the large number of people seeking care, and in the particular case of Colombia, the cost of health services [23, 24].

The objective of this study was to provide an overview on the main SRH issues affecting migrant Venezuelan women of reproductive age in Roraima, Brazil. The results should not only give insight into the prevailing SRHR issues in this migrant female population, but also provide an important contribution given the limited availability of SRHR data on this humanitarian crisis.

## Methods

### Study design

A large cross-sectional research study was conducted, combining both quantitative and qualitative data collection approaches. The quantitative study included face-to-face interviews with migrant Venezuelan women and the results are presented here. The qualitative results from the focus group discussions and the quantitative and qualitative findings on gender-based violence will be reported separately.

### Study tools

The Minimum Initial Service Package (MISP) readiness assessment tools from the Inter-Agency Working Group (IAWG) on Reproductive Health, adapted for use in Brazil [25], were used in the data analysis (Supplementary material). The Ethics Committee of the University of Campinas, Campinas, Brazil approved the study protocol and all participants signed an informed consent form before being interviewed.

### Data collection

The study was conducted in two cities in Roraima, where the principal border crossing points between Venezuela and Brazil are located: Boa Vista, the state capital, and Pacaraima, located at the main land crossing points from Venezuela. Roraima is a small state with a population of 600,000 inhabitants, located in north-western Brazil and sharing borders with Venezuela and Guiana. Boa Vista has 400,000 inhabitants and Pacaraima 17,000. According to the Brazilian constitution, healthcare is considered an obligation of the state and the right of all individuals (nationals, residents and migrants, including non-legally documented persons). Healthcare is provided by the National Health Service (*Sistema Unificado de Saúde, SUS*) and this is the principal source of healthcare for 75% of the entire population [26]. The SUS offers full medical and surgical care within the public healthcare network at no cost to patients, including prescribed medication in accordance with a list of essential medicines, and contraceptives. However, the only long-acting reversible contraceptive (LARC) method available is the TCu380A intrauterine device (IUD).

In Boa Vista, there is only one public maternity hospital providing services in Obstetrics and Gynaecology. Due to the increasing number of migrant Venezuelan women the number of deliveries has increased over recent years. The proportion of deliveries performed for Venezuelan migrants at that hospital increased from 3.4% of all deliveries in Boa Vista in 2016 to 26.1% in 2019 [27, 28].

### Study participants

This paper is based on analysis of the quantitative data collected from non-indigenous and indigenous women. The indigenous participants were from the *Warão* and *Ñ E Pa* tribes of the Orinoco Delta region; however, these participants were all fluent Spanish speakers. Venezuelan women aged 18–49 years, living in the cities of Boa Vista and Pacaraima in Roraima, Brazil, were included in the study. The participants were living in five of the eleven UNHCR shelters based in Boa Vista and in the two shelters based in Pacaraima; however, women living in informal non-UN settlements in Boa Vista who attended the *St. Agostinho* church to receive free food and goods from a programme supported by UNICEF and Caritas International were also included. Indigenous women not fluent in Spanish and adolescents under 18 years of age were excluded. In Boa Vista, a sample was purposively selected from the five largest UN shelters. These included the shelters with the highest proportion of women aged 18–59 years, and a shelter designated for indigenous persons. In Pacaraima, two additional shelters (including one exclusively for indigenous persons) established by the UNHCR were selected.

A total of 405 women were interviewed, representing 21.1% of the entire female population of the 13 UNHCR shelters in the two cities, with 343 women (84.7%) being recruited from UNHCR shelters (including 47 indigenous women) and 62 (15.3%) from the *St Agostinho* church. All the interviews were conducted in Spanish between 24 and 30 of November 2019. A team of five trained interviewers (three women and two men) performed the data collection. A pre-tested, semi-structured, electronic questionnaire was used that included both open-ended and closed-ended questions on sociodemographic characteristics (age, race, cohabitation status, education, employment, income, place of residence and migration information), pregnancy and childbirth (births and pregnancies, current pregnancy status, antenatal (ANC) or postnatal care and complications), other SRH issues (family planning preferences, other gynaecological issues), and availability of and access to SRH services, including user satisfaction. Data were entered directly into tablets and saved in a secure online database at the University of Campinas. The data entry system included validation rules to minimise data entry errors during the survey. A unique pre-defined identification number was attributed to each woman.

Migrant women living at UNHCR shelters or elsewhere have access to SRH services free of charge exclusively within the public healthcare network, including primary healthcare units and hospitals. At the UNHCR settlement, a mobile health unit visits periodically to check vaccination and ANC cards and to encourage pregnant women to attend ANC. All women give birth at the only public maternity hospital in Boa Vista. Normally, women in labour are transported to the hospital using transport services paid by the UN, the Brazilian army or other organisations. Reversible contraceptives are available free of charge only within the healthcare system and it is very difficult for migrant women to obtain postpartum or interval tubal ligation. At the UN shelters, a small room is set aside for emergencies and in some cases doctors or nurses affiliated to UNICEF or humanitarian organisations provide some healthcare. In addition, the Brazilian army has health teams and mobile units that periodically visit the shelters, providing primary medical care, including vaccination.

### Study sampling

Sample size was calculated to determine whether the SRH needs of migrant Venezuelan women are being adequately met in Roraima. It was assumed that around 50% of the women would not be receiving adequate contraceptive care. Calculation of the population size was based on information provided by the UNHCR, showing a total of 7233 persons living in the 13 UNHCR-administered shelters, including 1917 women aged 18–59 years, in the two towns during the data collection period. Consequently, it was estimated that, for a 95% confidence interval (95%CI) (Z = 1.96), a margin error of 5% (0.05) and an estimated response rate of 80%, at least 358 women would be required [24]. This number was increased to 405 women, including 47 from the indigenous population. Since the results were similar for the indigenous and non-indigenous women, the data were pooled together.

### Statistical analysis

Data analysis consisted of simple frequency distribution, using means and standard deviations (SD), as well as bivariate analyses using the χ^2^ or Fisher’s exact test and the calculation of 95% confidence intervals (CI). When the distribution of the data was not normal, the Mann-Whitney test for two independent groups was used. Univariate and multivariate analysis was performed (with *Stepwise* criteria of variables selection) in order to identify variables associated to lack of procurement for healthcare services. Significance level was set at *p* < 0.05.

## Results

Table 1 summarises the baseline characteristics of the study participants. Mean age (± SD) was 30.1 ± 8.6 years (range 18–49 years; 95%CI: 28.39–31.87). Most of the women were identified as biracial (62.0%), and most had a partner at the time of the interview (66.2%). Most had high school or some higher education (65.1%). Furthermore, 359/405 (88.6%) migrated from less than 1000 km away. Many of the women interviewed (75.6%) reported that they had migrated with children.

**Table 1:** Sociodemographic characteristics of the migrant Venezuelan women interviewed at the Brazilian-Venezuelan border (*n* = 405), 2019

Characteristics of the women	n (%)
*Age (years) (n = 405)*	
18–19	36 (8.9)
20–29	186 (45.9)
30–39	114 (28.2)
40–49	69 (17.0)
*Ethnicity (n = 405)*	
White	83 (20.5)
Indigenous	45 (11.1)
Biracial	251 (62.0)
Black or Asian	26 (6.4)
*Cohabitation status (n = 403)*	
Living with a partner	268 (66.2)
Without a partner	135 (33.3)
*Educational level (n = 405)*	
Illiterate	9 (2.2)
Primary school	132 (32.6)
Secondary school	180 (44.4)
Higher education	84 (20.7)
*Parity (n = 405)*	
0	67 (16.5)
1–2	154 (38.0)
3–5	154 (38.0)
6 or more	30 (7.4)
*Place of residence (n = 405)*	
UN shelter	343 (84.7)
Not in an official shelter	62 (15.3)
*Main reasons for migrating (n = 405)*	
Lack of work opportunities	326 (80.5)
Health problems	40 (9.9)
Violence	9 (2.2)
Other	30 (7.4)
*Employment status before migrating (n = 405)*	
Formal job	200 (49.5)
Informal job	97 (23.9)
Housewife or other	108 (26.6)
*Current employment status (n = 405)*	
Unemployed	378 (93.3)
Formal or informal employment	27 (6.7)

Table 2 describes the main SRH issues and concerns reported by the women. Two-thirds of those interviewed had an unmet family planning need and also reported a range of gynaecological complaints and symptoms of sexually transmitted infections, reflecting the finding that around two-thirds had consulted for SRH care and ANC care. However, the main issues concerned access to ANC consultations. Of 63 women (15.5%) who reported having given birth after arriving in Roraima, 15 had received no ANC, principally due to lack of access (10/15); however, some (5/14) considered that they did not need care. Ten women (15.9%) reported having delivery by Caesarean delivery; and 10 (15.9%) reported complications following childbirth (mainly fever, bleeding and hypertension). Forty-three women (68%) received no postnatal care, principally resulting from lack of access (18/43) and lack of information regarding the importance of postnatal care (20/43). Furthermore, 31 women (49.2%) reported that healthcare professionals failed to offer contraception following childbirth.

**Table 2:** Sexual and reproductive health issues and healthcare sought by migrant Venezuelan women at the Brazilian-Venezuelan border (*n =* 405), 2019

*Sexual and reproductive health issues and healthcare sought*	n (%)
*Main self-reported sexual and reproductive health concerns*	*N = 405*
Access to contraception	134 (33.1)
Gynaecological condition [e.g. menstrual disturbances, pelvic pain, vaginal discharge]	107 (26.4)
Symptoms related to a sexually transmitted infection	67 (16.5)
Prenatal or postnatal care	29 (7.2)
Gender-based violence	19 (4.7)
None	49 (12.1)
*Woman consulted for SRH issue (n = 405)*	
Yes	255 (63.0)
No	150 (37.0)
*Reasons for consultation (n = 255)*	
Contraception	79 (30.9)
Gynaecological problem	68 (26.7)
Prenatal care	45 (17.6)
Other	63 (24.7)

Table 3 summarises the main issues pertaining to access to SRH services and use. The majority of women who had received ANC stated that they were satisfied with the care received, which was adequate and included assessment of their weight, blood pressure, and abdominal circumference, as well as urine and blood tests. In some cases, an ultrasound scan was provided and advice was given on alert signs. Intrapartum care at the maternity hospital was considered excellent and the women were satisfied, particularly because they were allowed to have a companion with them during labour, an uncommon practice in Venezuela.

**Table 3:** Access to and use of sexual and reproductive health (SRH) services among migrant Venezuelan women at the Brazilian-Venezuelan border (*n* = 405), 2019

Variables	
Are you satisfied with the attention you received at the healthcare unit? (n = 247*)	
Satisfied	93 (37.7)
Partially satisfied	92 (37.2)
Dissatisfied	62 (25.1)
For those who stated that they were dissatisfied or partially satisfied, what was the reason? (n = 147*)	
Many people attending the unit	83 (56.5)
Did not like the care received	25 (17.0)
Long waiting time	18 (12.2)
Others	21 (14.3)
*What organisations do you know that provide SRH services? (n = 405)*	
Primary healthcare unit	293 (72.3)
Maternity hospital	44 (10.9)
Health post at the UN shelter	9 (2.2)
Don’t know any	59 (14.6)
Travel time from respondent’s place of residence to the healthcare unit providing SRH services (n = 333*)	
15 min	268 (80.5)
60 min	38 (11.4)
90 min	27 (8.1)
*Since arriving at the place of residence, which items of hygiene or childbirth have you received? (n = 405)*	
Disposable sanitary pads	269 (66.5)
Reusable cloth sanitary pads	3 (0.7)
Obstetric delivery kit	33 (8.1)
Personal hygiene items	27 (6.7)
None	73 (18.0)

*Not all of the women answered these questions

However, around 25% of the women indicated that they were dissatisfied with the care provided at the different healthcare units. This was primarily attributed to overcrowding at these units, which in many cases obliged women to come to the healthcare unit more than once to obtain a consultation (although this survey was conducted before the Covid-19 pandemic). Another source of dissatisfaction concerned women requiring contraception. Although contraceptives are provided free of charge within the public healthcare service, of the 79 women requesting contraception, 50 (63.3%) reported that they were unable to obtain the contraceptive method of their choice and 40 (50.6%) reported that they were unable to access any form of contraception. Injectable contraceptives, subdermal implants (requested by 31.2% of the women seeking contraception) and IUDs (requested by 20.8% of the women) were unavailable). Women reported no coercion regarding whether or not to use contraceptives.

Furthermore, the univariate analysis showed that migrant women who did not procure SRH services were indigenous, those who migrated with ≥4 relatives and women who were not pregnant during the interviews and women who did not have a delivery after migration. The multivariate analysis showed that the lack of procurement for SRH services was significantly associated with race (indigenous women; 4.6 times higher) and those that were not pregnant or did not have a delivery after migration (2.1 times higher).

Conversely, many women (75.4%) reported adequate access to personal hygiene kits and 94% of the women (*n* = 308/328) stated that these kits were distributed predominantly by multilateral international organisations; however, 203/323 of the women (62.8%) stated that the hygiene kits distributed were insufficient to meet their daily needs.

## Discussion

Our paper aimed to provide an overview of the main SRH issues and concerns affecting migrant Venezuelan women in Roraima, Brazil, with results showing that the main issues reported by these migrant women concerned unmet family planning needs and healthcare during pregnancy and following childbirth. Access to good quality SRH services is a basic human right and has the potential to save lives; nevertheless, these findings show that the provision of adequate SRH services to this population of migrant Venezuelan women is lacking. This was particularly true with respect to meeting family planning needs (use of and access to contraception), ability to use the required SRH services and access to ANC and postnatal care. Furthermore, the women’s ability to access healthcare and receive help to resolve these issues was very limited. Bearing in mind that most of the women interviewed in this study were actually residing in UN shelters, this finding suggests that such needs may be considerably greater among migrant women living in informal settlements.

Most of these migrant women were young, had migrated with a partner and children, and had more than 9 years of formal education. Although 50% reported having had a formal job before migration, almost all reported that they were currently unemployed in Brazil. This is despite the fact that the women interviewed reported that their main reasons for immigrating to Brazil were lack of opportunities and the prevailing health problems in Venezuela. Migrants cannot be legally employed in Brazil unless they have documentation proving that they are legal residents in the country. Furthermore, they need documentation before they can move to another state with a larger population where the possibilities of obtaining formal employment are greater.

Regarding unmet family planning needs, access to LARC methods was particularly poor and this was a major concern of many women in this study. Women stated that their inability to access LARCs prevents their family planning needs from being met and increases the likelihood of an unplanned pregnancy, which continues to pose an important challenge for them given the reality of their daily life. However, the SRH situation for migrant women is similar to that of many Brazilian women. In Brazil, the prevalence of LARC use is only 1.9% in women of reproductive age [29]. This has often been directly associated with the high prevalence of unplanned pregnancy in the country [30]. Although the copper IUD is generally available at all Brazilian public healthcare facilities, the prevalence of its use is low, while, implants and the hormonal intrauterine system are only available in a few public healthcare facilities.

In line with these findings, it is equally important to emphasize that these SRH issues faced by migrant Venezuelan women are an improvement on the situation of migrant Venezuelan women in Colombia, where they have to pay for medical care [31]. The free provision of SRH care to migrant women, including the provision of ANC, intrapartum and postnatal care, vaccinations and contraceptives by the SUS, is an important action to protect women and infants, and is considered an ethical one. In addition, the cost to donors/governments of providing feminine hygiene products is significant and other possibilities such as the provision of menstrual cups should be considered in an attempt to reduce this cost. Conversely, in the United Kingdom, the decision by the National Health Service establishing that migrants and those not ordinarily resident in the country should have to pay for ANC, intrapartum and postnatal care has been considered a violation of bodily autonomy and a health risk to women and neonates [32].

According to the Brazilian constitution, all citizens have the right to free healthcare within the SUS. Venezuelan migrants in Brazil enjoy similar health rights and full access without charge to the public healthcare system [33]. Yet, it is an important point that in Roraima, the state in which the largest number of these women is concentrated, the migrant population uses the same publicly funded programmes as the general population, and these programmes are facing severe shortages of healthcare providers, materials, medicines, contraceptives, tests and equipment. These shortages are directly attributed to the fact that both the municipal and state healthcare systems have failed to adequately prepare for the large numbers of migrants received; consequently, healthcare resources at health facility level are crippled because of the inadequate distribution of healthcare providers and resources to meet this increased demand. For example, the number of Venezuelan migrant women giving birth at the Boa Vista public maternity hospital increased from 288 in 2016 to 2875 in 2019, representing 3.4 and 26.1%, respectively, of all the deliveries in the city for the same years [27, 28]. This limited capacity of the healthcare system poses important risks for all women including migrants.

Access to the healthcare system is also associated with satisfaction. When evaluating the overall satisfaction of women with the SRH services provided, these migrant women reported that they were either satisfied or partially satisfied with the care they received at the healthcare facilities, particularly in relation to the ANC, intrapartum or postpartum care received. This finding is not surprising, as it is a longstanding tradition in Brazil to provide adequate care to pregnant women and children. The close proximity of the UN shelters to the primary healthcare units could also have helped increase satisfaction with prenatal care.

During the current SARS-CoV-2 pandemic, access to SRH services and use of these services by this migrant population is also expected to suffer [34] and this could impact the migrant Venezuelan population in the state of Roraima. Notwithstanding, maintaining the essential SRH services for this migrant population of women is pivotal if their pressing SRH needs are to be met [35].

The phenomenon of this migration from Venezuela to other Latin American countries poses important SRH challenges in addition to the burden of communicable diseases [36]. Ensuring access to the MISP and principally to the essential SRH services within the MISP, as indicated above, as part of the SARS-CoV-2 response could present a unique opportunity in this population of migrant women. Indeed, provision of these much needed services could also help contain the spread of the pandemic in this migrant population [37, 38].

In summary and in line with these results, to be able to respond more responsibly to the SRH challenges identified here these findings need to be shared to make the relevant authorities aware of the health priorities of these migrant women and to address the potential barriers associated with access to care in order to fortify SRH services. This should be conducted in parallel with appropriate sensitisation and mobilisation of all multilateral organisations, policy makers and stakeholders to save time and resources and avoid the risks of stigmatisation that could further prevent migrants from obtaining timely access and coverage for necessary healthcare, including SRH. There are still important gaps to be filled in guaranteeing girls’ and women’s rights, with migrant women in particular being the most vulnerable group.

### Strengths and limitations

The strengths of our study include the fact that it is the first to provide an overview of the status of SRHR issues and concerns among migrant Venezuelan women of 18 to 49 years of age in Brazil, including the availability and delivery of services, barriers to service uptake and related challenges in Brazil. The adequate sample size and selection permit generalisability of these findings to the larger population of migrant Venezuelan women in Brazil. The unwillingness of the women to disclose sensitive information related to SRH practices, service utilisation and health facility records constitutes a limitation of the study.

## Conclusions

National health systems must follow a needs-driven agenda that is responsive not only to the needs of the general population but also of this migrant Venezuelan population. Adequately coordinated efforts that include governmental, academia, non-governmental and international organisations constitute a crucial step to respond in a timely and appropriate manner to contribute to better access and service provision for this migrant population within the Brazilian National Health System.

## Supplementary Information

**Additional file 1.** Questionnaire ““EVALUATION OF THE SITUATION OF VENEZUELAN MIGRANT WOMEN IN THE STATE OF RORAIMA, BRAZIL”. Questionnaire applied to women during the interviews.

## Data Availability

The datasets used and/or analysed during the current study are available from the corresponding author on reasonable request.

## Abbreviations

ANC: Antenatal care
CI: Confidence Interval
HIV: Human Immunodeficient Virus
IAWG: Inter-Agency Working Group
IUD: Intrauterine device
LARC: Long-acting reversible contraception
MISP: Minimum Initial Service Package
PAHO: Pan American Health Organisation
SRHR: Sexual and reproductive health and rights
SRH: Department of Sexual and Reproductive Health and Research
SUS: *Sistema Unificado de Saúde*
UNHCR: United Nations High Commissioner for Refugees
UNICEF: United Nations Funds for Child
WHO: World Health Organisation
