# Supplementary material for: Maternal health among Venezuelan women migrants at the border of Brazil
Source: BMC Public Health. 2020 Nov 23;20:1771. doi: 10.1186/s12889-020-09912-x (PMC7682772; doi:10.1186/s12889-020-09912-x)
Supplement: Supplementary file 1 — Additional file 1. Questionnaire ““EVALUATION OF THE SITUATION OF VENEZUELAN MIGRANT WOMEN IN THE STATE OF RORAIMA, BRAZIL”. Questionnaire applied to women during the interviews. [file 12889_2020_9912_MOESM1_ESM.docx]

**“EVALUATION OF THE SITUATION OF VENEZUELAN MIGRANT WOMEN IN THE STATE OF RORAIMA, BRAZIL”**

**DATE OF INTERVIEW**: ____/____/________ **Code of interviewer:** |___|

**Initial of the woman´s name:** |___|___|___|___| **Number of research**:|___|___|___| ___| ***(do not fill in)***

**Place of interview: _____________________ Time of the interview:** |___|___|h |___|___|min

**SOCIO-DEMOGRAPHIC IDENTIFICATION**

**1. Place where woman is living:** UN shelter [ 1 ] Informal shelter [ 2 ] UN Indigenous shelter [ 3 ] Homeless [ 4 ] Other [5 ], If other, specify **__________________________(Textual)**

**2. Date of born (d/m/y):** |___|___/___|___|/___|___|___|___| **2A. Age:** |___|___| years

**3. Race:** White [ 1 ] Black [ 2 ] Biracial [ 3 ] Indigenous [ 4 ] Asian [ 5 ]

**4. Marital status:** Married [ 1 ] with a partner [2 ] Without a partner [3] Divorced [4] Widowed [5]

Prefer not mention [6]

**4.A If married or with a partner, Do you live together at the moment?** Yes [ 1 ] No [ 2 ]

**5. Do you write and read?** Yes [ 1 ] No [ 2 ] **6. Do you go to school?** Yes [ 1 ] No [ 2 ]

**7. What is you educational level (completed):** Primary level [ 1 ] High School [ 2 ] University [ 3 ]

No educational level [ 4 ] Did not answer/ Do not know [ 5 ]

**8. Whats is your religion?** Catholic [1] Protestant [2] Pentecostal [3] Other Christian [4] Other [5]

Do not have [ 6 ] Prefer do not mention [ 7 ]

**9. Do you remember date you arrived in Brazil?** |___|___/___|___| (month/year) Do not remember [88]

**10. Why did you migrated to Brazil?** Insecurity [ 1 ] corruption [2 ] domestic violence [3]

social violence [4] no economic opportunity [5 ] prefer do not answer [ 6 ] Other [ 7 ] (Specify)_______________________________**(Text)**

**11. How many family member/persons have migrated with you?: |___|___|**

**12. Who migrated with you?** Partner Yes [ 1 ] No [ 2 ]; Son/daughter Yes [ 1 ] No [ 2 ] **;**  Parents Yes [ 1 ] No [ 2 ]; Brothers/sisters Yes [ 1 ] No [ 2 ]; Other Yes [ 1 ] No [ 2 ]

**13. Do you have son or daughter with you in Brazil?** Yes [ 1 ] No [ 2 ] *If no, please go to Q15*

**13.A. How many?** |___|___| **13.B What are the age?** |___|___| ; |___|___| ; |___|___| ; |___|___|

**14. If any son/daughter under 6 years old, are they in the school?** Yes[ 1 ] No [ 2 ] **14.A All of them?**

Yes [ 1 ] No [ 2 ]

**15. Do you have other son/daughter who stayed in the Venezuela?** Yes [ 1 ] No [ 2 ] **15.A. How many? |___|___|**

**16. Where do you live in Venezuela before immigrating to Brazil? ______________________________ (Textual)**

**17. What do you do/work in Venezuela before migrated to Brazil? __________________________________(Textual)**

**18. Do you work now?:** Yes [ 1 ] No [ 2 ] If no, please go to Q 20

**If Yes, do you receive a salary for your occupation?** Yes [ 1 ] No [ 2 ]

**19. What is your current occupation?** Formal [ 1 ] Informal [ 2 ]

**20. What is the familiar income:** ________________R$.

**20.A What is the main familiar income source?** Your job Yes[ 1 ] No[ 2 ];

Your partner job Yes[ 1 ] No[ 2 ]; Assistance Yes [ 1 ] No [ 2 ]; Other Si [ 1 ] No [ 2 ]

**PREGNANCIES AND DELIVERY ASSISTANCE**

**21.** **Have you been pregnant ?** Yes[ 1 ] No [ 2 ] If NO, please go to Q44

**22. If yes,** Gravidity_____Deliveries (vaginal and Casearean) _____Abortions_____

**23. After you arriving in Brazil, do you have a delivery? Yes** [ 1 ] No [ 2 ] If NO, please go on Q31

**24. Where do you get assistance?** Primary health Unit |___|; Women Referral Center |___|; Maternity Hospital|___|; Other (Specify)_______________________________(**Textual)**

**25.** **How was your delivery?** Vaginal Delivery [ 1 ] Forceps [ 2 ] Caesarean [ 3 ]

**26**. **Do you have any delivery complication?**  Yes [ 1 ] No [ 2 ] If *NO, please go to Q 27*

**If, YES, what was your delivery complication?** Bleeding [ 1 ] Fever/infection [ 2 ] High blood pressure [ 3 ] Breastfeeding [ 4 ] Other (Specify) [ 5 ] **____________________________(Textual)**

**26.B Did you schedule an appointment for the problem?** Yes [1] No [ 2 ]

*If NO,*

**26.C If you did not schedule an appointment, why?** Did not consider important [ 1 ]

Did not know where to go [ 2 ] Did not look for a service [ 3 ] Did not have Access/ the Access to health unit was difficult [ 4 ] Did not like the attention received [ 5 ] Did not have time [ 6 ] Other (Specify) [ 7 ] If other, **____________________(Textual)**

**27. Did you receive antenatal care during your last pregnancy?**  Yes [ 1 ] No [ 2 ]

*If NO, please go to Q28*

**27.A If Yes, ¿Where did you do antenatal care?** Health Unit |___|; Women Referral Center |___|; Maternity Hospital|___|; Other (Specify)_______________________________(**Textual)**

*If NO,*

**28. Why did not you receive antenatal care?** Did not consider important [ 1 ]

Did not know where to go [ 2 ] Did not look for a service [ 3 ] Did not have access/ the access to health unit was difficult [ 4 ] Did not like the attention received [ 5 ] Did not have time [ 6 ] Other (Specify) [ 7 ] If other, **____________________(Textual)**

**29. Did you receive the postnatal care?**  Yes [ 1 ] No [ 2 ] *If NO, please got to Q30*

**29.A.** **How many visits?**  |___|___|

**29.B. If YES, Where did you receive the postnatal care?** Health Unit |___|; Women Referral Center |___|; Maternity Hospital|___|; Other (Specify)_______________________________(**Textual)**

**29.C. Was any contraceptive method offered to you in postnatal care:**

Yes [1] No [2] Did not know [3]

**30. If NO, Why did not you receive postnatal care?** Did not consider important [ 1 ]

Did not know where to go [ 2 ] Did not look for a service [ 3 ] Did not have Access/ the Access to health unity was difficult [ 4 ] Did not like the attention received [ 5 ] Did not have time [ 6 ] Other (Specify) [ 7 ] If other, **____________________(Textual)**

**31. Are you pregnant at the moment?** Yes [ 1 ] No [ 2 ] Do not know/are not secure about it [ 2 ]

*If NO or DO NOT KNOW, please go to Q44*

**32.** **If YES, Do you know the gestational age?** |___|___|weeks or **months of pregnancy?** |___| **months** Did not apply /Do not know [ 8 ]

**33. Do you know where will should go to delivery?** Health Unit |___|; Women Referral Center |___|; Maternity Hospital|___|; Other (Specify)_______________________________(**Textual)**

**34. Are you going to the antenatal care? Yes** [ 1 ] No [ 2 ] If NO, please go to *Q 38*

**35. If YES, where are you going to?** Health Unit |___|; Women Referral Center |___|; Maternity Hospital|___|; Other (Specify)_______________________________(**Textual)**

**36. How many antenatal visits did you go until now?** |___|___| Did not apply /Do not know [ 8 ]

**37.We want to know if:**

**37.A. Did they measure your weight? Yes** [ 1 ] No [ 2 ]

**37.B. Were they measure your blood pressure? Yes** [ 1 ] No [ 2 ]

**37.C.** **Are they measure the waist circumference**  **Yes** [ 1 ] No [ 2 ]

**37.D.** **Did you do the urine exam?**  **Yes** [ 1 ] No [ 2 ]

**37.E. Did you do blood exam?**  **Yes** [ 1 ] No [ 2 ]

**37.F.** **Did you do ultrasound?**  **Yes** [ 1 ] No [ 2 ]

**37.G. Were you oriented about the alert signs?**  **Yes** [ 1 ] No [ 2 ]

**38. If NO, Why did not you do antenatal care appointment?** Did not consider important [ 1 ]

Did not know where to go [ 2 ] Did not look for a service [ 3 ] Did not have Access/ the Access to health unity was difficult [ 4 ] Did not like the attention received [ 5 ] Did not have time [ 6 ] Other (Specify) [ 7 ] If other, **____________________(Textual)**

**39. During this current pregnancy, did you have any health problems?** Yes [1] No [ 2 ] ]

If No, please go to Q44

**39.A. If you answered YES, tell us which was the problem?** Bleeding [1] Fever/infection [ 2 ] High blood pressure [3] Breastfeeding [4] Other (Specify) [5] **____________________________(Textual)**

**39.B Did you schedule an appointment for the problem? Yes** [1] No [ 2 ]

*If NO,*

**39.C If you did not schedule an appointment, why?** Did not consider important [ 1 ]

Did not know where to go [ 2 ] Did not look for a service [ 3 ] Did not have Access/ the Access to health unity was difficult [ 4 ] Did not like the attention received [ 5 ] Did not have time [ 6 ] Other (Specify) [ 7 ] If other, **____________________(Textual)**

**OTHER TOPICS ABOUT SEXUAL AND REPRODUCTIVE HEALTH**

**44. Which are the main concerns you have about sexual and reproductive health (SRH)?**

Contraception [1] Pregnancy/Pregnancy Monitoring [2]; Postpartum/Postpartum Monitoring [3].

Gynaecological problem [4] Sexually transmitted disease [5] Victim of violence [6] Other [7] (Specify)

Textual______________________________________________________________________________

**45. Have you already gone to a Health Unit to seek care for yourself? Yes [ 1 ] No [ 2 ]**

**46. What was the reason? For contraception [ 1 ]; Antenatal control [ 2 ]; Postnatal control [ 3 ] Gynaecological problem [ 4 ] Victim of violence [ 5 ] .**

**Other Health Care [5], If Other (Specify) _______________________(Textual)**

*If the reason was NOT to seek contraception, please go to Q53*

**47. If the reason was to seek contraception, did you get what you wanted? Yes [ 1 ] No [ 2 ]**

**48. If "NO"; did you get a different contraceptive than what you were looking for? Yes [ 1 ] No [ 2 ]**

**49. What did you want for birth control?**

Copper IUD [1] Hormonal IUD [2] Implant [3] Injectable [4] Pills [5] Tubal ligation [6]

**50. Have you ever been coerced to use birth control? Yes [ 1 ] No [ 2 ]**

**51Have you ever been coerced to NOT use birth control? Yes [ 1 ] No [ 2 ]**

**52. From whom? ________________________________________________________(Textual)**

**53. Do you consider the care received at the Health Unit satisfactory? Yes [ 1 ] No [ 2 ]**

**54. If you answered NO, what is the reason? Language barrier [ 1 ] No privacy [ 2 ] Did not feel comfortable with the health care provider [ 3 ] Consultation was too fast [ 4 ] Too many people [ 5 ] Waiting time is too long [ 6 ] No place to wait to be seen/no place to sit [ 7 ] Other [ 8 ] (Specify) _________________________________________________(Textual)**

**55. If you have been a victim of sexual or physical violence please tell us:**

**56. Did you report it to any authorities? Yes [ 1 ] No [ 2 ] Prefer not to answer [ 3 ]**

**57. Were you well received? Yes [ 1 ] No [ 2 ] Prefer not to answer [ 3 ]**

**58. Did you receive the assistance you were looking for? Yes** [ 1 ] No [ 2 ] Prefer not to answer [ 3 ]

**SEXUAL AND REPRODUCTIVE HEALTH SERVICES**

**59.** **Are you generally satisfied with the care you have received in SRH since you migrated to Brazil? Satisfied [ 1 ] Partially satisfied [ 2 ] Not satisfied [ 3 ] N/A [ 4 ]**

**60. What place do you know that provide SRH services or health care for women?**

Health Unit |___|; Women Referral Center |___|; Maternity Hospital|___|; Other (Specify)_______________________________(**Textual)**

**61. How far are these facilities from where you are staying?**

**|___|___|min |___|hours**

**62. Have you ever attended a programme specifically for young people? Yes [ 1 ] No [ 2 ]**

**63. Since your arrival, what menstruation supplies, hygiene or birth kits were distributed to women or girls in this shelter or area?**

**Disposable sanitary pads [ 1 ] Tampons [ 2 ] Clothing/reusable sanitary pads [ 3 ] Birth kits [ 4 ] Other [ 5 ] If other (Specify) __________________________________________ (Textual)**

**64. Who did the distribution, where was it done and how many times was it done in the last month?**

**_________________________________________________________________(Textual)**

**65. What did the community think about these distributions?**

**_________________________________________________________________(Textual)**

**66. What reasons have you heard for not having these supplies?**

**_________________________________________________________________(Textual)**

**67. Who could you discuss with if there are unmet needs of women/girls in this shelter/area (e.g. shelter leader, informal shelter leader, women's association, NGO, UN, etc.)?**

**_________________________________________________________________(Textual)**

**Anything else you want to add to this interview?**

**_____________________________________________________________(Textual)**

**Interviewer, thanks the lady!**

**End time of interview: |___|___|h |___|___|min**
